# Supplementary material for: m5C methylated lncRncr3–MeCP2 interaction restricts miR124a-initiated neurogenesis
Source: Nat Commun. 2024 Jun 15;15:5136. doi: 10.1038/s41467-024-49368-w (PMC11180186; doi:10.1038/s41467-024-49368-w)
Supplement: Supplementary file 1 — Supplementary Information [file 41467_2024_49368_MOESM1_ESM.pdf]

m^{5}C methylated *lncRncr3*–MeCP2 interaction restricts *miR124a*-initiated neurogenesis

Jing Zhang[1]*, Huili Li[1] and Lee A. Niswander[1]*

[1]Department of Molecular, Cellular, and Developmental Biology. University of Colorado Boulder, Boulder; CO 80309; USA

*Authors for correspondence:

jing.i.zhang@colorado.edu; Kinnzhang@gmail.com

lee.niswander@colorado.edu

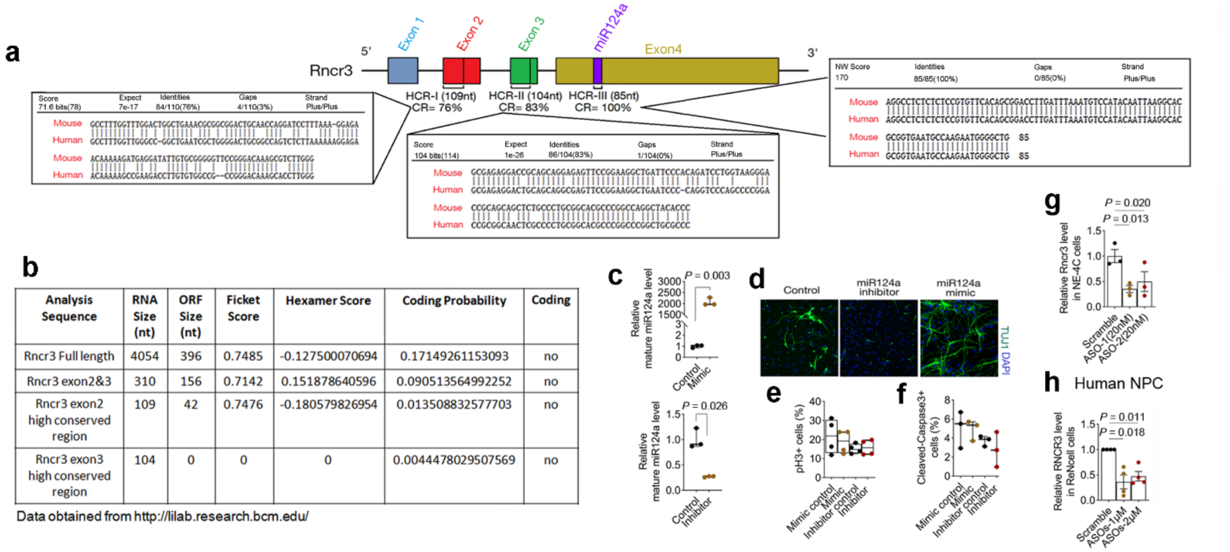

Analysis Sequence	RNA Size (nt)	ORF Size (nt)	Ficket Score	Hexamer Score	Coding Probability	Coding
Rncr3 Full length	4054	396	0.7485	-0.127500070694	0.17149261153093	no
Rncr3 exon2&3	310	156	0.7142	0.151878640596	0.090513564992252	no
Rncr3 exon2 high conserved region	109	42	0.7476	-0.180579826954	0.013508832577703	no
Rncr3 exon3 high conserved region	104	0	0	0	0.0044478029507569	no

Supplementary Fig. 1 | *Rncr3* exons 2/3 are conserved and knock down of this lncRNA in mouse and human NEPCs/NPCs. a, Schematic diagram and nucleotide sequence of *Rncr3* exon/intron structure showing three highly conserved regions between human and mouse: exon 2 (76% over 109 bp; 64% over the entire 191 nt of exon2), exon 3 (83% over 104 bp; 78% over the entire 120 nt of exon3) and exon 4 (100% over 85 bp). **b,** Assessment of coding potential of full length *Rncr3* or *Rncr3* exon 2 and exon 3 using coding potential assessment tool (CPAT), a logistic regression model that predicts coding probability from nucleotide sequences. These sequences are predicted to be non-coding. **c,** NE-4C cells were transfected with *miR124a* mimic oligonucleotide to increase activity or a blocking oligonucleotide to decrease the level of mature *miR124a*. **d,** NE-4C cells grown under differentiation conditions assessed for neuronal differentiation by TUJ1 staining following treatment with *miR124a* inhibitor or mimic oligo. DAPI stains nuclei (blue). **e, f,** Effect of *miR124a* inhibitor or mimic oligo on the percentage of pH3 (**e**) and cleaved-Caspase3 (**f**) positive cells under proliferation conditions. In **c**, **e** and **f**, boxplots show all data points with box which extends from the 25th to 75th percentiles and whiskers showing min to max, and the line in the middle of the box is plotted at the median, Student's *t*-test, two-sided. In **c**, n=3 biologically independent experiments; in **e**, n=4 biologically independent experiments; in **f**, n=3 biologically independent experiments. **g,** Knock-down (KD) efficiency of Antisense oligo (ASO) against *Rncr3* or scramble control in proliferating NE-4C cells (RT-qPCR

for exons 2 to 3; Student's *t*-test, mean ± s.e.m.; n=3 biologically independent experiments). **h,** *RNCR3* KD efficiency in human ReNcell CX cells with 1 μM or 2 μM pooled ASOs transfected twice and assayed 24 hours after 2nd transfection(Student's *t*-test, mean ± s.e.m.; n=4 biologically independent experiments). Source data are provided as a Source Data file.

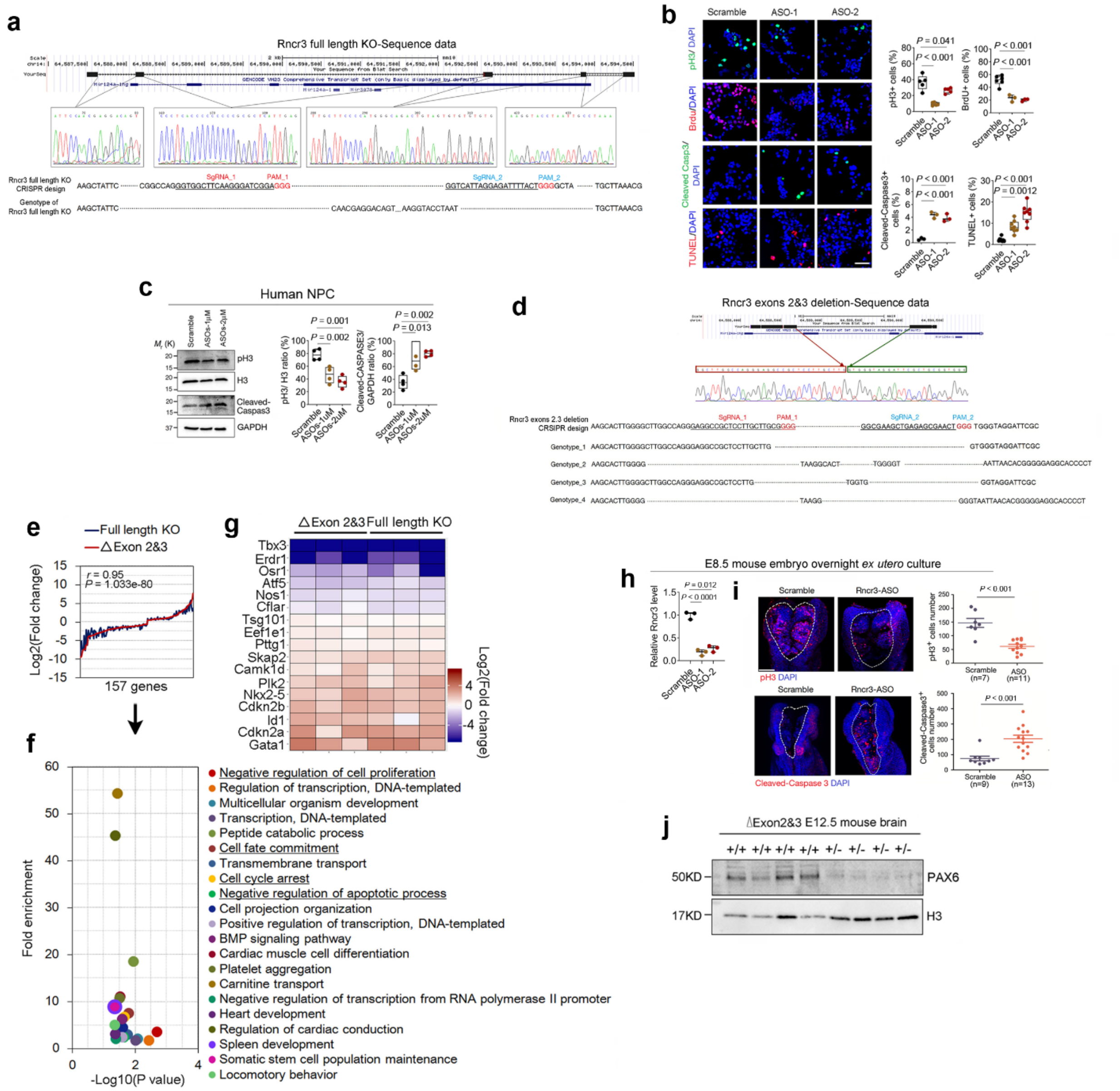

Supplementary Fig. 2 | CRISPR mediated regional deletion of *Rncr3*, knock down of *Rncr3* in cells and RNA-seq data show *Rncr3* exons 2/3 independently maintain NEPCS/NPCs.

a, Genotyping-PCR and sequencing data of isolated clones generated by CRISPR-Cas9 gene editing for *Rncr3* full-length deletion. Off-target effect analysis of *Rncr3* full-length deletion CRISPR sgRNAs is shown in Source data for **Supplementary Fig.2a**. **b,** *Rncr3* ASO KD in NE-4C cells stained for proliferation markers (pH3, n=5 biologically independent experiments and BrdU, n=3) and apoptotic markers (cleaved-Caspase3, n=3 and TUNEL, n=8). Scale bar,

50 µm. One-way ANOVA. **c,** Representative western blot and quantification for pH3 and cleaved-Caspase3 following *RNCR3* KD in human ReNcell CX cells (n=4 biologically independent experiments). Student's *t*-test, two-sided. **d,** Genotyping-PCR and sequencing data of isolated NE-4C clones generated by CRISPR-Cas9 gene editing for *Rncr3* exons 2&3 deletion. Off-target effect analysis of *Rncr3* exons 2&3 deletion CRISPR sgRNAs is shown in Source data for **Supplementary Fig. 2d**. **e,** RNA-seq data from exons 2/3 deletion and full length KO NE-4C cells under proliferation conditions show 157 genes whose expression is consistently changed relative to wildtype (Pearson's r = 0.95; P = 1.033e-80). **f,** Gene Ontology (GO) function analysis of 157 genes shows biological process enrichment for regulation of cell proliferation and apoptosis (underlined; $P < 0.05$). **g,** Heat map presents fold change of enriched genes functioning in proliferation and cell survival in three different clones of each genotype. **h,** *In vivo* KD efficiency of ASOs against *Rncr3* or scramble control in wild-type mouse embryos cultured for 12 hours. Student's *t*-test, two-sided; n=3 biologically independent experiments. **i,** *Rncr3* exons 2/3 ASO KD in wildtype mouse embryos cultured from ~E8.5 to E9.0 (dorsal views, dotted lines outline neural folds). Immunofluorescence images and quantification for pH3 (scramble n=7 embryos, ASO n=11 embryos) and cleaved-Caspase3 positive cells (scramble n=9 embryos, ASO n=13 embryos) relative to scramble control ASO. Student's *t*-test, two-sided, mean ± s.e.m. **j,** Decreased expression of the NPC marker PAX6 protein in *Rncr3* exons 2/3 deletion heterozygous E12.5 mouse brain. In **b, c**, **h**, **i**, Boxplots show all data points with box which extends from the 25th to 75th percentiles and whiskers showing min to max, and the line in the middle of the box is plotted at the median. Source data are provided as a Source Data file.

a

	Rncr3-exon1 expression	Rncr3-exon2/3 expression	Rncr3-miR124a region expression	Mature miR124a-3P expression
WT	1	1	1	1
△Exon 2&3 Clone_1	1.6301441	0.0061722	2.0279189	7.801897
△Exon 2&3 Clone_2	1.0352646	0.0123444	2.7702189	21.64492
△Exon 2&3 Clone_3	3.29436301	0.0086086	3.6807506	14.01067
△Exon 2&3 Clone_4	3.0844207	0.0051187	3.8370565	24.25938

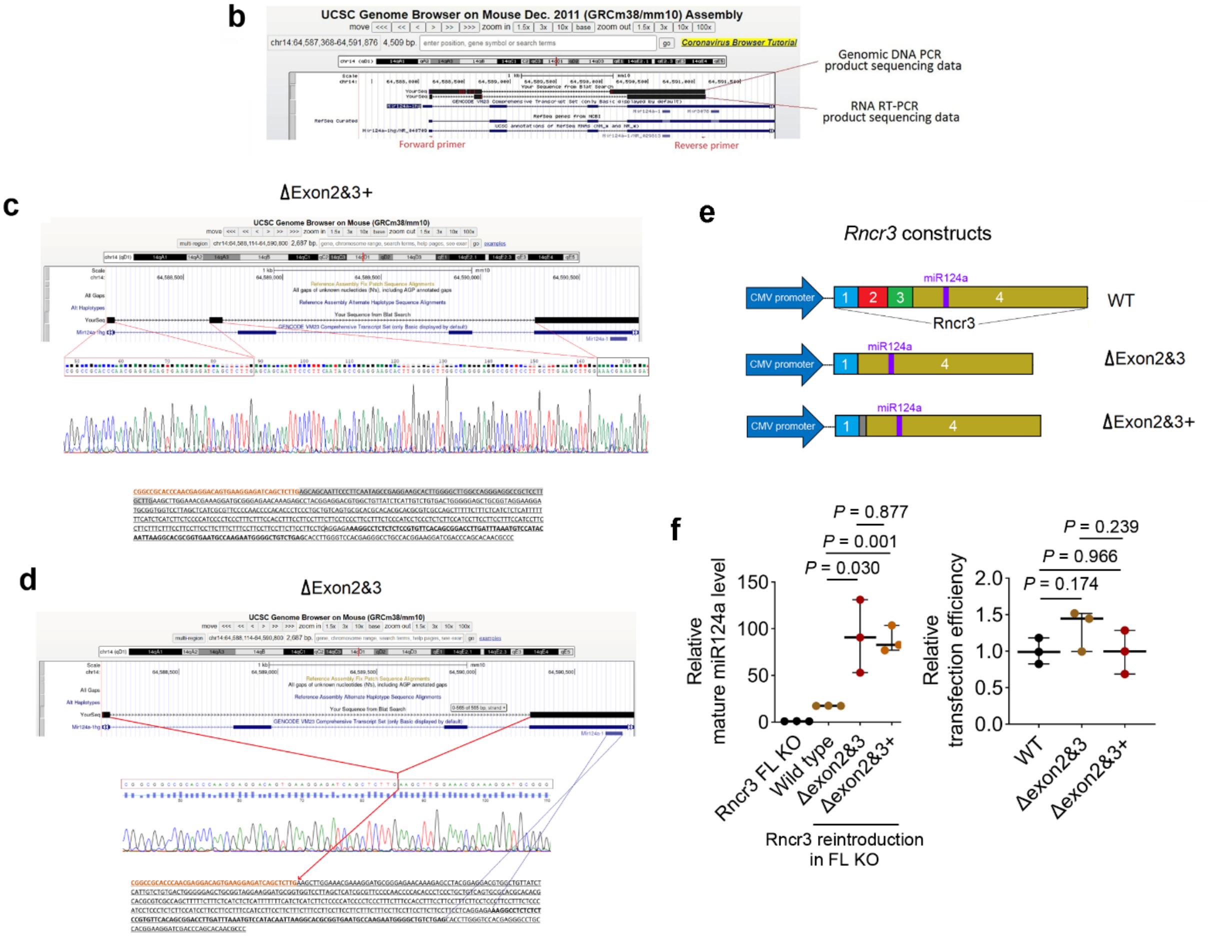

Supplementary Fig. 3 | *Rncr3* exons 2/3 regulate *miR124a* production. a, RT-qPCR data for relative levels of transcription in regions of exon 1, exons 2/3 and exon 4 (*miR124a* region) and mature *miR124a* in exons 2/3 deletion and wildtype NE-4C cells under proliferation conditions. **b,** RT-PCR and sequencing of transcripts derived from *Rncr3* locus in exons 2/3 deletion cells showing generation of a spliced transcript with exon 1 and exon 4 including the *miR124a* sequences. In the exon 2&3 deletion line, the resulting RNA transcript includes a cryptic exonic fragment between exons 1 & 4. **c-e,** To clarify any negative or positive effects of this cryptic exon on *miR124a* biogenesis, we created different plasmid constructs to express wildtype

Rncr3, **c,** *Rncr3* exon2/3 deletion with the cryptic exon (Δexon2&3+) or **d,** *Rncr3* exon2/3 deletion without the cryptic exon region. **e,** schematic of the plasmid constructs. **f,** Plasmids were transfected into *Rncr3* KO NE-4C cells to assay the level of *miR124a* production. RT-qPCR data show that under similar transfection efficiency, full-length *Rncr3* (containing exons 2&3) has limited *miR124a* expression (17.5 fold over background in the KO); while the two plasmids of *Rncr3* Δexon2&3 and Δexon2&3+ induced *miR124a* by 92-fold and 88-fold, respectively. This indicates the cryptic exon does not alter functionality. Student's *t*-test, two-sided; n=3 biologically independent experiments; boxplots show all data points with box which extends from the 25th to 75th percentiles and whiskers showing min to max, and the line in the middle of the box is plotted at the median. Source data are provided as a Source Data file.

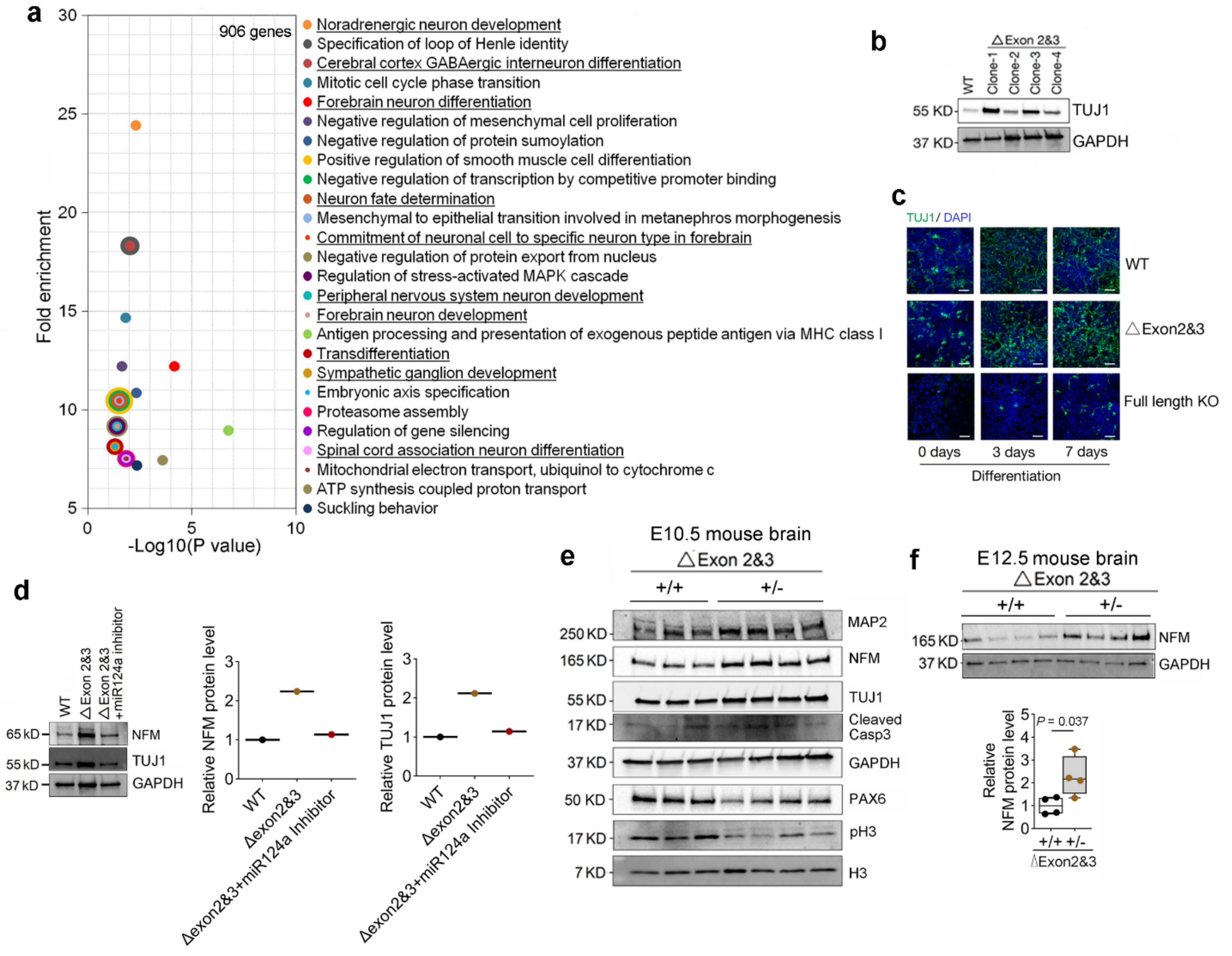

Supplementary Fig. 4 | *Rncr3* exons 2/3 regulate neuronal differentiation. a, In **Fig. 3a**, a total of 906 genes were upregulated in exons 2/3 deletion cells (red dots) but not changed in full length deletion (blue dots) in RNA-seq data. Here is shown the Gene Ontology (GO) Biological Process (BP) analysis of the 906 genes indicating enrichment for neuronal differentiation (underlined). **b,** Immunoblot image shows increased neuronal marker TUJ1 protein levels in *Rncr3* exons 2/3 deletion NE-4C cells under proliferation conditions. **c,** Immunofluorescent staining of TUJ1 in *Rncr3* exons 2/3 deletion and *Rncr3* full-length deletion cells under proliferation conditions or 3 and 7 days under neuronal differentiation conditions. **d,** Immunoblot data of exons 2/3 deletion NE-4C cells treated with *miR124a* inhibitor shows attenuation of NFM and TUJ1 levels. **e,** Immunoblots of proteins isolated from *Rncr3* exons 2/3 deletion heterozygous mouse embryo brains (E10.5) detected for neuronal differentiation markers MAP2, NFM, and TUJ1, apoptosis marker (cleaved-Caspase3), and NEPC markers PAX6 and pH3. **f,** Immunoblot and quantification data show increased NFM protein levels in E12.5 *Rncr3* exons 2/3 deletion

heterozygous mouse embryo brains compared with wildtype. Student's *t*-test, two-sided, boxplots show all data points with box which extends from the 25th to 75th percentiles and whiskers showing min to max, and the line in the middle of the box is plotted at the median. n = 4 embryos. Source data are provided as a Source Data file.

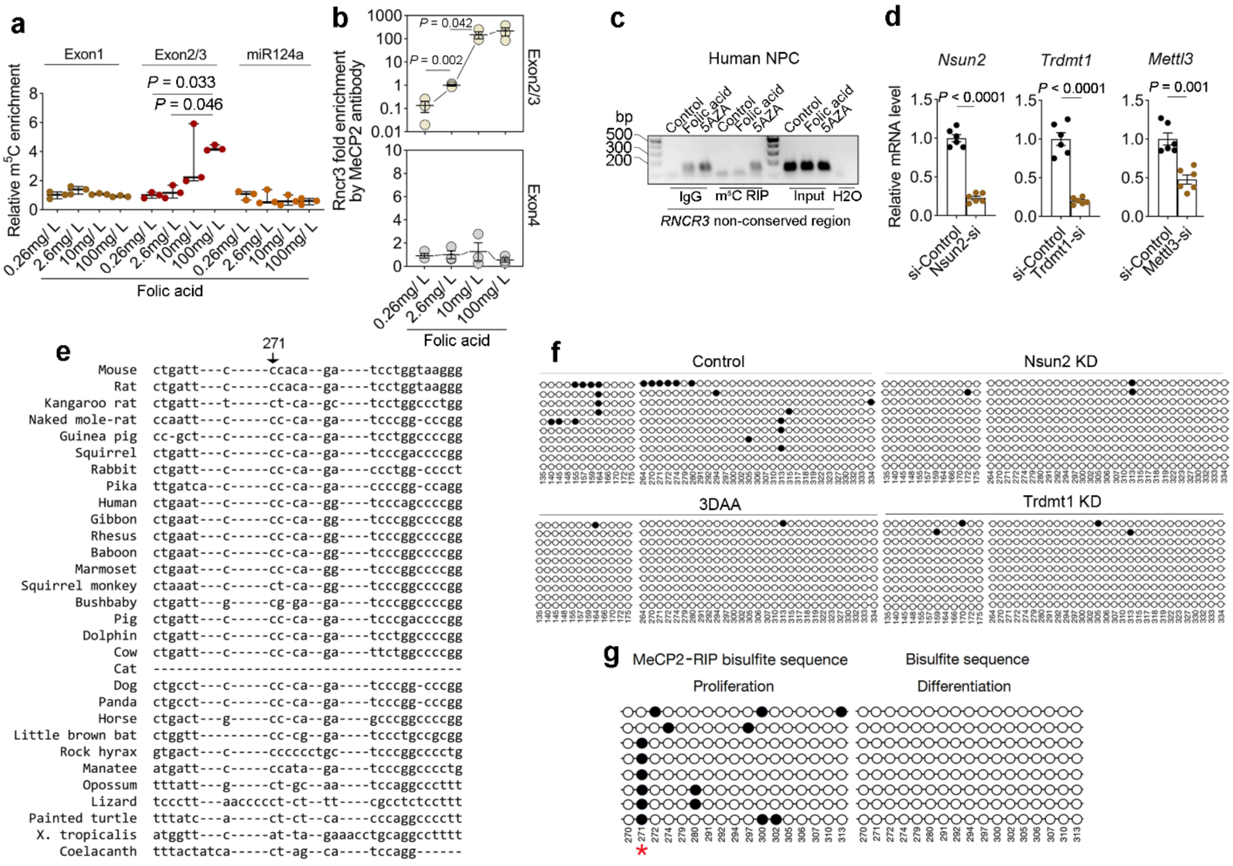

Supplementary Fig. 5 | MeCP2 protein binds methylated *Rncr3* exons 2/3. a, Anti-5-methylcytosine antibody used to capture methylated RNAs (m^5C-RIP) followed by probing for *Rncr3* exons in proliferating NE-4C cells treated with increasing levels of folic acid. Student's *t*-test, two-sided, n=3 biologically independent samples. Boxplots show all data points with box which extends from the 25th to 75th percentiles and whiskers showing min to max, and the line in the middle of the box is plotted at the median. **b,** Relative enrichment of *Rncr3* exons 2/3 or exon 4 regions by MeCP2-RIP in response to increasing levels of folic acid. Student's *t*-test, two-sided, mean ± s.e.m., n=3 biologically independent samples. **c,** In **Fig. 4d** (right panel), m^5C-RIP followed by RT-qPCR shows in ReNcell CX cells that RNCR3 conserved region (Sequences in *Rncr3* exon3 shown in **Supplementary Fig. 1a**) is methylated and with increased methylation in response to folic acid treatment or greatly decreased methylation by 5AZA treatment. By contrast, here the RT-qPCR gel electrophoresis data show that *RNCR3* non-conserved region (3′ distal expression region of *RNCR3*) is not significantly methylated. **d,** Quantitative RT-PCR showing efficiencies of siRNA KD of *Nsun2*, *Trdmt1*, and *Mettl3* in NE-4C cells. Student's *t*-test, two-sided, mean ± s.e.m., n=6 biologically independent samples. **e,** Genomic sequence alignment of a portion of *Rncr3* exon 3 showing conservation in mammals

(downloaded from the UCSC genome browser). Cytosine 271 is indicated by an arrow. **f,** RNA bisulfite sequencing of total RNA isolated from NE-4C cells cultured under proliferation, 3DAA-, *Nsun2* knockdown-, and *Trdmt1* knockdown- conditions. The methylation status of individual cytosine in *Rncr3* exons 2 and 3 in independent clones (vertical, n=10) is shown for each treatment group with black circles indicating methylated cytosine residues, and white circles indicating unmethylated cytosine residues. **g,** Under proliferation conditions (left panel), MeCP2 RIP-pulldown and bisulfite sequencing identified individual methylated cytosines (black circles) and unmethylated cytosines (white circles) in *Rncr3* exon 3 (nucleotides 270–313 containing 17 cytosines, n=8 independent clones vertically aligned). Red star denotes frequently methylated cytosine residue, C271. Under differentiation conditions (right panel), bisulfite sequencing of *Rncr3* locus from total RNA shows an absence of cytosine methylation in exon 3 region (summary data from 50 individual cDNA clones).

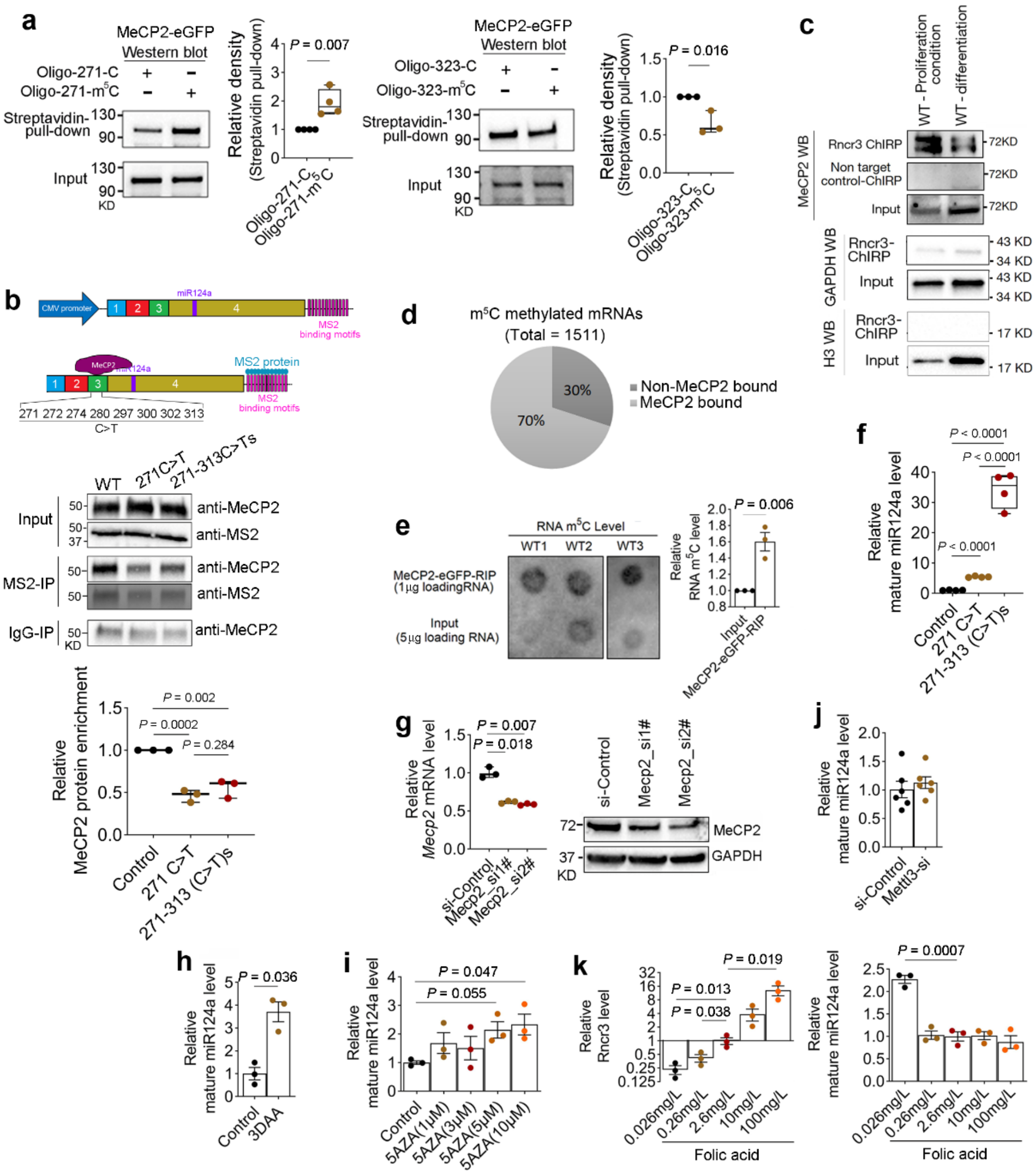

Supplementary Fig. 6 | MeCP2 protein binds methylated *Rncr3* exons 2/3 to regulate *miR124a* production. a, Biotin-labeled oligo-271-m^{5}C RNA oligonucleotide was used for RNA pulldown followed by immunoblotting for MeCP2-eGFP-WT protein transfected into NE-4C cells. Biotin-labeled RNA oligonucleotides NC-Oligo-323-m^{5}C as negative control was used for RNA pulldown experiments followed by immunoblotting for MeCP2-eGFP-WT protein transfected into NE-4C cells (n=4 biologically independent experiments). **b,** Top, Schematic of the method for purification of protein-*Rncr3* complexes using MS2-tagged Rncr3. The mRNAs of wild-type *Rncr3* (or 271C>T, or 271-313 Cs>Ts) bearing the MS2 binding hairpins is recognized by the

fusion protein of MS2-GFP, which allows for mRNA–protein complex purification using anti-MS2 antibody-conjugated beads. Bottom, western blotting and quantitative data show *Rncr3* RNA-binding to MeCP2 for the different genotypes (n=3 biologically independent experiments). **c,** ChIRP assays in NE-4C for *Rncr3* enrichment of MeCP2 protein under proliferation or neuronal differentiation conditions (non-target control: LacZ-antisense DNA probes). Control experiments for ChIRP assays shown by immunoblotting with antibodies against GAPDH and H3 proteins. **d,** An in-depth analysis based on two published databases[1,2] suggests that in the mouse brain, 70% of m^5C-modified mRNAs were also bound by MeCP2. The details of MeCP2 bound m^5C-modified genes list are found in Source data for **Supplementary Fig. 6d**. **e,** Dot blot analysis of m^5C levels following enrichment for MeCP2-bound RNAs (MeCP2-eGFP-RIP of NE-4C cells) showed significantly higher m^5C levels in MeCP2 protein-bound target RNAs than that of Input control without eGFP-antibody enrichment (n=3 biologically independent experiments). **f,** Mature *miR124a* levels after plasmid transfections of *Rncr3* KO cells with wildtype *Rncr3*, *Rncr3* 271C>T, or 271-313Cs>Ts (n=4 biologically independent experiments). **g,** *Mecp2* KD in NE-4C cells. Quantitative RT-PCR assays (left) and immunoblotting (right) using antibodies against MeCP2 or GAPDH proteins to show KD efficiency of two different siRNAs in NE-4C cells (n=3 biologically independent experiments). In **a**, **b**, **f** and **g**, Student's *t*-test, two-sided, boxplots show all data points with box which extends from the 25th to 75th percentiles and whiskers showing min to max, and the line in the middle of the box is plotted at the median. **h.** Levels of mature *miR124a* in NE-4C cells treated with 3DAA under proliferation conditions. (n=3 biologically independent samples). **i,** Effect of increasing doses of 5AZA treatment on mature *miR124a* expression in NE-4C cells (n=3 biologically independent samples). **j,** Levels of mature *miR124a* in NE-4C cells treated with siRNA against *Mettl3*-si under proliferation conditions. (n=6 biologically independent samples). **k,** Relative expression levels of *Rncr3* and mature *miR124a* in response to increasing doses of folic acid under proliferation conditions of NE-4C cells (n=3 biologically independent samples). In **e** and **h-k**, Student's *t*-test, two-sided, mean ± s.e.m. Source data are provided as a Source Data file.

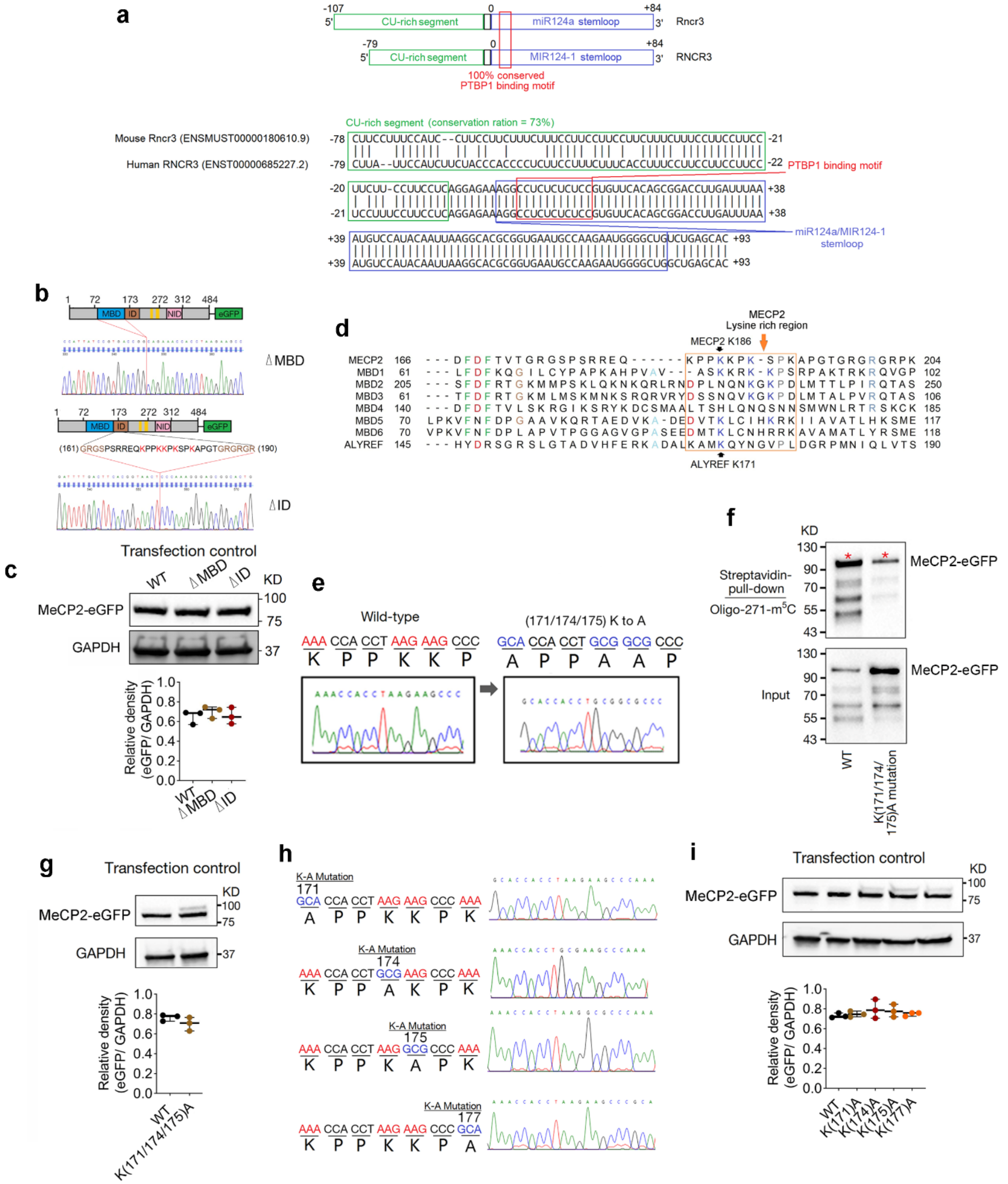

Supplementary Fig. 7 | MeCP2 recognition of cytosine methylated *Rncr3* RNA through conserved lysine residues. a, Schematic diagram and sequence comparison of the region around mouse *miR124a*/human *MIR124-1*. The 107 nucleotide CU-rich region previously identified[3], a shows strong conservation between mouse *Rncr3* and human *RNCR3* (79 nt, conserved identity 73%). Notably, a completely conserved PTBP1 binding motif

"CCUCUCUCUCC" is closely adjacent to the 5’ of *miR124a*/*MIR124-1* stem–loop in mouse and human pri-*miR-124-1.* **b,** Schematic diagram of MeCP2 deletion proteins. MBD is the defined cytosine-methylated DNA binding domain, NID is the NCoR/SMRT interaction domain and ID is intervening domain. **Fig. 6a** shows the thirty amino acid sequence of the ID containing the two RG repeat motifs and lysine (K)-rich region. RG repeat motifs are identified as RNA binding modules[4,5]. We generated a full-length MeCP2 construct with MBD deletion or 30 amino acid ID deletion. **c,** Equivalent transfection efficiency of MeCP2 constructs into NE-4C cells with MBD deletion or ID deletion. **d,** Multiple sequence alignments of MeCP2 (GenBank: NP_001104262.1) and MBD family members MBD1-6 (GenBank: NP_001191065.1, NP_003918.1, NP_001268382.1, NP_001263199.1, NP_060798.2, and NP_443129.3), and the sequence of ALYREF protein (GenBank: NP_005773.3), modified from[6]. **e,** Three lysine to alanine mutations in K171-K175 region (K171/174/175A). **f,** Immunoblotting (quantified in **Fig. 6d**) for association of biotin-labeled RNA Oligo-271-m^{5}C with nuclear lysates from NE-4C cells transfected with MeCP2 proteins. Red stars indicate the target pull-down bands. **g,** Equivalent transfection efficiency of MeCP2 wildtype and multiple lysine mutant constructs into NE-4C cells for RIP experiments as detected by immunoblotting using antibodies against GAPDH and eGFP proteins. **h,** Individual lysine to alanine mutations on residues of K171, K174, K175 and K177. **i,** As in (**g**), equivalent transfection efficiency of MeCP2 wildtype and individual mutant constructs into NE-4C cells for RIP experiments. In **c**, **g** and **i**, Student’s *t*-test, two-sided, n=3 biologically independent experiments. Boxplots show all data points with box which extends from the 25th to 75th percentiles and whiskers showing min to max, and the line in the middle of the box is plotted at the median. Source data are provided as a Source Data file.

Supplementary Table 1: Cell lines used in this study

Name	Company	Catalog No
Mouse: NE-4C cells	ATCC	#CRL-2925
Human: ReNcell CX immortalized cell line	Millipore	SCC007
Human: HEK 293 cells	ATCC	#CRL-1573

Supplementary Table 2: Chemicals used in this study

Name	Company	Catalog No
Folic acid	Sigma-Aldrich	# F7876
ATRA	Sigma-Aldrich	# 302-79-4
Puromycin	Thermo Fisher Scientific	# A1113803
TRIzol reagent	Thermo Fisher Scientific	# 15596018
11-digoxigenin UTPs	Roche	# 11209256910
Lipofectamine 3000	Thermo Fisher Scientific	# L3000015
Lipofectamine 2000	Thermo Fisher Scientific	# 11668027
5-azacytidine	Sigma-Aldrich	# A2385
3-deazaadenosine	Cayman	# 9000785
Pierce™ 16% Formaldehyde (w/v), Methanol-free	Fisher Scientific	# 28908
Sodium deoxycgolate	Sigma-Aldrich	# 30970
Urea	Sigma-Aldrich	# U5378
DEPC	Sigma-Aldrich	# D5758-25ML
Fragmentation Reagent	Ambion	# AM8740
Igepal CA-630	Sigma-Aldrich	# I8896-50ML
Vanadyl ribonucleoside complex	NEB	# S1402S
Formamide	Sigma-Aldrich	# F9037
2x Laemmli Sample Buffer	Bio-Rad	# 1610737
4–20% Mini-PROTEAN® TGX™ Precast Protein Gels	Bio-Rad	# 4561095
DTT	Sigma-Aldrich	# 43816

Supplementary Table 3: Peptides, and recombinant proteins used in this study

Name	Company	Catalog No
Phusion High-Fidelity DNA Polymerase (2 U/mL)	Thermo Fisher Scientific	# F530L
FailSafe™ Enzyme Mix Only	Lucigen Corporation	# FSE51100
FailSafe™ PCR 2X PreMix G	Lucigen Corporation	# FSP995G
Basic fibroblast growth factor	Millipore	# GF003
Epidermal growth factor	Millipore	# GF001
Laminin	Millipore	# CC095
Envigo Bioproducts Inc Rat Serum for Whole Embryo	Envigo	# NC0369840
LightCycler 480 SYBR Green I Master	Roche	# 04707516001
BSA	NEB	# B9000S
PureProteome™ Protein A/G Mix Magnetic Beads	EMD Millipore	# LSKMAGAG02
RNase inhibitor	NEB	# M0314L
Protease Inhibitor Cocktail	Abcam	# ab271306
DNase I	NEB	# M0303S
Streptavidin-conjugated magnetic beads	NEB	# S1420S
RNasin® Ribonuclease Inhibitor	Promega	# N2111

Supplementary Table 4: CRISPR/CAS9 system induced *Rncr3* regional deletions in NE-4C cells and mouse

Name	sgRNA
Rncr3_Exon2/3 deletion_sgRAN-1	GAGGCCGCTCCTTGCTTGCG
Rncr3_Exon2/3 deletion_sgRAN-2	GGCGAAGCTGAGAGCGAACT
Rncr3_Full length KO_sgRAN-1	GGTGGCTTCAAGGGATCGGA
Rncr3_Full length KO_sgRAN-2	GGTCATTAGGAGATTTTACT

Supplementary Table 5: Recombinant DNA used in this study

Name	Company	Catalog No
pGL3-U6-sgRNA-PGK-puromycin	Addgene	RRID: Addgene 51133
pCas9_GFP	Addgene	RRID: Addgene_44719
pEGFP-N1_MeCP2(WT)	Addgene	RRID: Addgene_110186
phage-CMV-CFP-24×MS2bs	Addgene	RRID: Addgene_40651
pMS2-GFP	Addgene	RRID: Addgene_27121
phage-CMV-Rncr3_WT-24×MS2bs	This study	
phage-CMV-Rncr3_271C_T-24×MS2bs	This study	
phage-CMV-Rncr3_271-313C_Ts-24×MS2bs	This study	
MeCP2 mutations_ MBD deletion	This study	
MeCP2 mutations_ ID deletion	This study	
MeCP2 mutations_(K171/174/175A)	This study	
MeCP2 mutations_(K171A)	This study	
MeCP2 mutations_(K174A)	This study	
MeCP2 mutations_(K175A)	This study	
MeCP2 mutations_(K177A)	This study	

Supplementary Table 6: Genotyping Primers for CRISPR/CAS9 system induced *Rncr3* regional deletions

Name	5'-3'
Exon2 deletion test_ Forward	ATAGCCGAGGAAGCACTTGG
Exon2 deletion test_ Reverse	TCCCACTCCCTACTAGTGCC
Exon3 deletion test_ Forward	CACCCGGACCCTACACTCAA
Exon3 deletion test_ Reverse	CCCGTGTTAATTACCCCGACT
Exon2/3 deletion test_ Forward	ACCCAACGAGGACAGTGAAG
Exon2/3 deletion test_ Reverse	GACAGGTAGGGATGAAGGTG
Rncr3 full length deletion test_ Forward (1)	CACCTTCCGCCATTCGTCT
Rncr3 full length deletion test_ Reverse (1)	GATTTCGGCTTCGAGTGCAT
Rncr3 full length deletion test_ Forward (2)	GACAAGCAGAAGGTGTGCAA
Rncr3 full length deletion test_ Reverse (2)	CCTCAGCAGCACTAGGCTATC
Mouse genotyping nested PCR_ Forward (1)	TCACCCAACGAGGACAGTGAAGGAGAT
Mouse genotyping nested PCR_ Reverse (1)	GACAGGTAGGGATGAAGGTG
Mouse genotyping nested PCR_ Forward (2)	ATAGCCGAGGAAGCACTTGG
Mouse genotyping nested PCR_ Reverse (2)	CTCAGACAGCCCCATTCTTG

Supplementary Table 7: Experimental models: Organisms/strains were used in this study

Name	Company	Catalog No
Mouse: C57BL/6J (B6) (WT)	The Jackson Laboratory	JAX: 000664
Mouse: FVB/N	The Jackson Laboratory	JAX:001800
Mouse: Rncr3 exons 2/3 deletion mice	This study	

Supplementary Table 8: Mouse ASOs for *Rncr3* knockdown

NO.	2′OMe/DNA chimera antisense oligonucleotides	GC%
1	5'-mA*mA*mG*mA*mC*G*C*T*T*T*G*T*C*C*C*mG*mG*mA*mA*mC-3'	55
2	5'-mG*mA*mA*mU*mC*A*G*C*C*T*T*C*C*G*G*mA*mA*mC*mU*mC-3'	55

Supplementary Table 9: Commercial kits used in this study

Name	Company	Catalog No
Quick-RNA^{TM} Miniprep Kit	Zymo Research	# R1055
First Strand cDNA Synthesis Kit	NEB	# E6300S
In Situ Cell Death Detection Kit	Roche	# 12156792910
RNA ChIP-IT Magnetic Chromatin Immunoprecipitation Kit	Active Motif	# 53024
EZ RNA Methylation Kit	Zymo Research	# R5002
Poly (A) polymerase tailing reactions kit	EpicentreBio	# PAP5104H
SMARTer™ RACE cDNA Amplification Kit	Clontech	# 634923 and 634924
QuikChange II Site-directed mutagenesis kit	Agilent	# 200521
3’mRNA-Seq Library Prep Kit (Universal Plus mRNA-Seq with NuQuant)	Tecan Genomics	# 0520

Supplementary Table 10: Primers for qRT-PCR

Name	Forward	Reverse
Rncr3 (Exon_1)	ACCCAACGAGGACAGTGAAG	AAGGCGGATCAAGAGCTGAT
Rncr3 (Exon_2/3)	CAACGCTGCCCAACAGTA	ATCTGTGGGAATCAGCCTTC
Rncr3 (miR_region)	AAGGCCTCTCTCTCCGTGTT	CTCAGACAGCCCCATTCTTG
Rncr3 (Exon_4)	CCTCCTTGGGGCTGTGGCTG	GAGCGATGGGGAGAGGCCCA
Pre-miR124a	AAGGCCTCTCTCTCCGTGTT	CTCAGACAGCCCCATTCTTG
Gapdh	GGGTTCCTATAAATACGGACTGC	CCATTTTGTCTACGGGACGA
Hprt	CCTGGTTCATCATCGCTAATC	TCCTCCTCAGACCGCTTTT
18S	GCAATTATTCCCCATGAACG	GGGACTTAATCAACGCAAGC
Malat1	TTTCAGAAACTTGACTTCGAACA	TTCGGTCTTCTGGCTCAAAT
miR124a_RT	GTCGTATCCAGTGCAGGGTCCGAGGTATTCGCACTGGATACGACGGCATT	
U6_RT	GTCGTATCCAGTGCAGGGTCCGAGGTATTCGCACTGGATACGACAAAATA	
miR124a_qPCR	GCCCCTAAGGCACGCGGT	
U6_qPCR	GCGCGTCGTGAAGCGTTC	
miR/U6_qPCR_ Universal Rev		GTGCAGGGTCCGAGGT
hRNCR3	AGGCCTCTCTCTCCGTGTTCA	CAGCCCCATTCTTGGCATTCAC
Mecp2_qPCR	CAGCTCCAACAGGATTCCAT	TCTTCTGACTTTTCCTCCCTGA
Nsun2_qPCR	TGGGTACAAAAGCCATGCCA	CCAGGCAAGTTCTTCAGGGT
Trdmt1_qPCR	TGGACCACGTATCTGTGCTG	GCATCCTCTGCAGCCTGTAA
Mettl3_qPCR	GGCGTGCAGAACAGGATTTG	CTTAGCCGGCTCCTTAGCTG
DROSHA, DGCR8 RIP-qPCR	AAGGCCTCTCTCTCCGTGTT	CTCAGACAGCCCCATTCTTG
hRNCR3-conserved region- qPCR	TCTACGTCCACCCTTTACCTG	CCTGGGGATTCAGCCTTC
hRNCR3-nonconserved region	CGTGATGCAAGCTTATATGATTCT	CCCCCAGAAGACTCCATTC

Supplementary Table 11: List of antibodies used in the study

Name	Catalog No	Application
Rabbit polyclonal anti-pH3	Millipore Sigma, #06-570, RRID:AB_310177	IF, 1:200; WB, 1:500
Rabbit polyclonal anti-H3	Abcam, #ab1791, RRID:AB_302613	WB, 1:14000
Rabbit polyclonal anti-Cleaved Caspase-3	Cell Signaling, #9661, RRID:AB_2341188	IF, 1:200; WB, 1:500
Rabbit polyclonal anti-Tuj1	Sigma-Aldrich, #T3952, RRID:AB_1841226	IF, 1:400; WB,1:4000
Rabbit polyclonal anti-Gapdh	Sigma-Aldrich, #G9545, RRID:AB_796208	WB, 1:5000
Mouse monoclonal anti-Map2	Sigma-Aldrich, # M4403, RRID:AB_477193	WB, 1:500
Mouse monoclonal anti-NFM-anti160kD	Abcam, #ab65845, RRID:AB_1139297	WB, 1:1000
Rabbit polyclonal anti-Ptbp1	Thermo Fisher Scientific, #PA5-81297, RRID:AB_2788516	RIP, 4 µg per RIP
Rabbit monoclonal anti-Drosha	Cell Signaling Technology, # 3364; RRID:AB_2238644	RIP, 4 µg per RIP
Rabbit polyclonal anti-Dgcr8	Thermo Fisher Scientific, # PA5-40122; RRID:AB_2606318	RIP, 4 µg per RIP
Mouse monoclonal anti-BrdU	Molecular Probes, # A-21304; RRID:AB_221472	IF, 1:200
Mouse monoclonal anti-MeCP2	Active Motif, # 61285; RRID:AB_2572268	RIP, 4 µg per RIP WB, 1:500
Mouse IgG2c, kappa monoclonal [18C8BC7AD10] - Isotype Control antibody	Abcam, # ab170191; RRID:AB_2861163	RIP, 4 µg per RIP
Rabbit IgG Control Antibody, Unconjugated	Sigma-Aldrich, # I5006; RRID:AB_1163659	RIP, 4 µg per RIP
Rabbit polyclonal anti-5-Methylcytosine (5-mC)	Active Motif, # 61255, RRID:AB_2783884	RIP, 2 µg per RIP
Rabbit polyclonal anti-eGFP	Thermo Fisher Scientific, # CAB4211, RRID:AB_10709851	WB, 1:500 RIP, 4 µg per RIP
Anti- MS2 Coat Protein	Millipore Sigma, # ABE76-I, RRID:AB_2827507	RIP, 4 µg per RIP

Abbreviation: IF, Immunofluorescence; WB, Western blot; RIP, RNA immunoprecipitation.

Supplementary Table 12: Primers to clone *Rncr3* for RNA *in situ* hybridization probes

Name	5'-3'
Forward	CGCGGCGATCGCGCAGTAATACGACTCACTATAGGGAGGATATTGTGCGGGGTTC
Reverse	ACGCGACGCGTGCGATTTAGGTGACACTATAGAACGCGATGAGCTAAGGACC

Supplementary Table 13: Human ASOs (Phosphorothioate antisense) RNCR3 knockdown in human NPC line

NO.	DNA antisense oligonucleotides (all Phosphorothioate)	GC%
1	5'-C*C*C*C*C*A*G*A*A*G*A*C*T*C*C*A*T*T*C*T*A-3'	52
2	5'-C*T*G*T*G*A*A*C*A*C*G*G*A*G*A*G*A*G*A*G*G-3'	57
3	5'-C*A*G*C*C*C*C*A*T*T*C*T*T*G*G*C*A*T*T*C*A*C-3'	55
4	5'-G*A*A*T*C*A*G*C*C*T*T*C*C*G*G*A*A*C*T*C-3'	54
Scramble	5'-C*C*T*T*C*C*C*T*G*A*A*G*G*T*T*C*C*T*C*C-3'	60

Supplementary Table 14: Biotinylated ChIRP probes (20nt)

NO.	Biotinylated ChIRP probes
1	5'-AAGGCGGATCAAGAGCTGAT-Bio-3'
2*	5'-AAGACGCTTTGTCCCGGAAC-Bio-3'
3*	5'-GAATCAGCCTTCCGGAACTC-Bio-3'
4	5'-TTTCGTTTCCAAGCTTTTTC-Bio-3'
5	5'-GGAACGCGATGAGCTAAGGA-Bio-3'
6	5'-GCTGTGAACACGGAGAGAGA-Bio-3'
7	5'-TGTTTGCTTCCTAGATTCTA-Bio-3'
8	5'-GACAGGTAGGGATGAAGGTG-Bio-3'
9	5'-GCACAATACAAGGTAGCTGC-Bio-3'
10	5'-TGCGCTGTCCATGAACAAAT-Bio-3'
11	5'-CTGTAGCTTCTGGATTTCTG-Bio-3'
12	5'-GCCTGCTTCAAAACAGACTT-Bio-3'
13	5'-GATGGAGACGGGATGGAACT-Bio-3'
14	5'-TAAACATCCTTCATGGGCTT-Bio-3'
15	5'-GAAACTAGGCCAGAGAGGTA-Bio-3'
16	5'-TGGCTAGCTCTAACATTCTC-Bio-3'
17	5'-CTTTCTGCCTGCAAGAGTAA-Bio-3'
18	5'-TATGGCGTCTCTTAGGACAG-Bio-3'
19	5'-CCCAAAATGGTACAGCACAC-Bio-3'
20	5'-GTAGAGGATTGCTAATGGGG-Bio-3'
21	5'-TTTGCGCTCTCAGAGCAAAA-Bio-3'
22	5'-CACACGCCAATAACACACGA-Bio-3'
23	5'-CTGAGCAGGTCATTAGAGTC-Bio-3'
24	5'-AGAGAAGGGCAGCGAATGTA-Bio-3'
25	5'-CCTTCATTTTCGCCATAAGA-Bio-3'
26	5'-AGTGGGGACTAAGGCAGTAG-Bio-3'
27	5'-TCTCTGACAGGGGAGAGAAA-Bio-3'
28	5'-AGTGATGGATACGGTTGTGT-Bio-3'
29	5'-GAATGGGGGTGGGTATTATG-Bio-3'
30	5'-TGACTCATGGGCTCAGAAAC-Bio-3'

"-Bio-3'" is noted as 3' end labeling by biotin.

Supplementary Table 15: Bisulfite treated RNA RT-PCR primers

Name	5'-3'
Bar-coded anchor RT primer	AAGCAGTGGTATCAACGCAGAGTACNNNT (30)VN, and V=A, G or C; N= A, C, G or T; "NNN" noted as bar-code
Nested PCR1st Round_Universal Primer Mix_long	CTAATACGACTCACTATAGGGCAAGCAGT GGTATCAACGCAGAGT
Nested PCR1st Round_Universal Primer Mix_short	CTAATACGACTCACTATAGGGC
Nested PCR2nd Round_Universal Primer	AAGCAGTGGTATCAACGCAGAGT
Rncr3_Exon2_methylation test_1	GCGGATTGTAATTAGGATTTTTTAAAGGAG
Rncr3_Exon2_methylation test_2	GGAGAATAAAAAGATGAGGATATTGTG
Rncr3_Exon3_methylation test_1	CGTAGTAGGAGAGTTTCGGAAGG
Rncr3_Exon3_methylation test_2	GGAGAGTTTCGGAAGGCTGATT

Supplementary Table 16: Primers for generating a construct that contains multiple lysine to alanine mutations in K171-K175 region (K171A/K174A/K175A) in MeCP2

Primer name	5'-3'
a511g_a512c_a520g_a521c_ a523g_a524c_ Forward	CTGGTTGGGAGATTTGGGCGCCGCAGGTGGTGCCTG CTCTCTCCTGGAGGGG
a511g_a512c_a520g_a521c_ a523g_a524c_ Reverse	CCCCTCCAGGAGAGAGCAGGCACCACCTGCGGCGC CCAAATCTCCCAACCAG

Supplementary Table 17: Primers for generating constructs that contain individual mutations of lysine to alanine (K171A, K174A, K175A, K177A) in MeCP2

**Primer name**	**5'-3'**
K171A_Mu_ Forward	CTGGTTGGGAGATTTGGGCTTCTTAGGTGGTGCCTGCTCTCT CCTGGAGGGG
K171A_Mu_Reverse	CCCCTCCAGGAGAGAGCAGGCACCACCTAAGAAGCCCAAAT CTCCCAACCAG
K174A_Mu_ Forward	CTGGTTGGGAGATTTGGGCTTCGCAGGTGGTTTCTGCTCTCT CCTGGAGGGG
K174A_Mu_Reverse	CCCCTCCAGGAGAGAGCAGAAACCACCTGCGAAGCCCAAAT CTCCCAACCAG
K175A_Mu_ Forward	CTGGTTGGGAGATTTGGGCGCCTTAGGTGGTTTCTGCTCTCT CCTGGAGGGG
K175A_Mu_Reverse	CCCCTCCAGGAGAGAGCAGAAACCACCTAAGGCGCCCAAAT CTCCCAACCAG
K177A_Mu_ Forward	CTGGTTGGGAGATGCGGGCTTCTTAGGTGGTTTCTGCTCTCT CCTGGAGGGG
K177A_Mu_Reverse	CCCCTCCAGGAGAGAGCAGAAACCACCTAAGAAGCCCGCAT CTCCCAACCAG

Supplementary Table 18: Primers for generating constructs that contain mutation(s) of exon3 in Rncr3

**Primer name**	**5'-3'**
Rncr3_ Mu_271C-T_Forward	CAGCAGGAGAGTTCCGGAAGGCTGATTCTCACAGATC CTGGTAAGGGAC
Rncr3_ Mu_271C-T_Reverse	GTCCCTTACCAGGATCTGTGAGAATCAGCCTTCCGGA ACTCTCCTGCTG
Rncr3_ Mu_(271_313)C-T_Forward	AGGCTGATTCTTATAGATCTTGGTAAGGGACCGCAGTA GTTTTGCCCTGCGGTACGCCCGGCCA
Rncr3_ Mu_(271_313)C-T _Reverse	TGGCCGGGCGTACCGCAGGGCAAAACTACTGCGGTC CCTTACCAAGATCTATAAGAATCAGCCT

Supplementary References:

1 Maxwell, S. S., Pelka, G. J., Tam, P. P. L. & El-Osta, A. Chromatin context and ncRNA highlight targets of MeCP2 in brain. *Rna Biol* **10**, 1741-1757 (2013). https://doi.org:10.4161/rna.26921

2 Amort, T. *et al.* Distinct 5-methylcytosine profiles in poly(A) RNA from mouse embryonic stem cells and brain. *Genome Biol* **18**, 1 (2017). https://doi.org:10.1186/s13059-016-1139-1

3 Yeom, K. H. *et al.* Polypyrimidine tract-binding protein blocks miRNA-124 biogenesis to enforce its neuronal-specific expression in the mouse. *Proc Natl Acad Sci U S A* **115**, E11061-E11070 (2018). https://doi.org:10.1073/pnas.1809609115

4 Tillotson, R. *et al.* Radically truncated MeCP2 rescues Rett syndrome-like neurological defects. *Nature* **550**, 398-401 (2017). https://doi.org:10.1038/nature24058

5 Good, K. V., Vincent, J. B. & Ausio, J. MeCP2: The Genetic Driver of Rett Syndrome Epigenetics. *Front Genet* **12**, 620859 (2021). https://doi.org:10.3389/fgene.2021.620859

6 Yang, X. *et al.* 5-methylcytosine promotes mRNA export-NSUN2 as the methyltransferase and ALYREF as an m(5)C reader. *Cell Res* **27**, 606-625 (2017). https://doi.org:10.1038/cr.2017.55
