# Supplementary material for: m5C methylated lncRncr3–MeCP2 interaction restricts miR124a-initiated neurogenesis
Source: Nat Commun. 2024 Jun 15;15:5136. doi: 10.1038/s41467-024-49368-w (PMC11180186; doi:10.1038/s41467-024-49368-w)
Supplement: Supplementary file 3 — Reporting Summary [file 41467_2024_49368_MOESM3_ESM.pdf]

nature portfolio

Corresponding author(s): Lee A. Niswander

Last updated by author(s): Apr 21, 2024

Reporting Summary

Nature Portfolio wishes to improve the reproducibility of the work that we publish. This form provides structure for consistency and transparency in reporting. For further information on Nature Portfolio policies, see our Editorial Policies and the Editorial Policy Checklist.

Statistics

For all statistical analyses, confirm that the following items are present in the figure legend, table legend, main text, or Methods section.

n/a	Confirmed	
☐	☒	The exact sample size (*n*) for each experimental group/condition, given as a discrete number and unit of measurement
☐	☒	A statement on whether measurements were taken from distinct samples or whether the same sample was measured repeatedly
☐	☒	The statistical test(s) used AND whether they are one- or two-sided<br>*Only common tests should be described solely by name; describe more complex techniques in the Methods section.*
☐	☒	A description of all covariates tested
☐	☒	A description of any assumptions or corrections, such as tests of normality and adjustment for multiple comparisons
☐	☒	A full description of the statistical parameters including central tendency (e.g. means) or other basic estimates (e.g. regression coefficient) AND variation (e.g. standard deviation) or associated estimates of uncertainty (e.g. confidence intervals)
☐	☒	For null hypothesis testing, the test statistic (e.g. F, t, r) with confidence intervals, effect sizes, degrees of freedom and P value noted<br>*Give P values as exact values whenever suitable.*
☒	☐	For Bayesian analysis, information on the choice of priors and Markov chain Monte Carlo settings
☒	☐	For hierarchical and complex designs, identification of the appropriate level for tests and full reporting of outcomes
☐	☒	Estimates of effect sizes (e.g. Cohen's *d*, Pearson's *r*), indicating how they were calculated

Our web collection on statistics for biologists contains articles on many of the points above.

Software and code

Policy information about availability of computer code

Data collection	Imaging was performed on a Nikon A1 confocal microscope. RNA-seq was run on the Illumina NovaSEQ6000 system
Data analysis	IMARIS 8.0.2 and ImagJ 1.53c were used to perform quantification analysis. Statistical analysis was performed using SPSS (25.0.0) and GraphPad Prism 9.0.0 (121). GraphPad Prism 9.0.0 (121) was utilized for data visualization. With RNA-seq data, differential expression was evaluated using deseq2 (version 3.14)86 within Rstudio (version 1.4.1103) utilizing R (version 4.1.2), the data was visualized by Rstudio (version 3.5.1) and excel.

For manuscripts utilizing custom algorithms or software that are central to the research but not yet described in published literature, software must be made available to editors and reviewers. We strongly encourage code deposition in a community repository (e.g. GitHub). See the Nature Portfolio guidelines for submitting code & software for further information.

Data

Policy information about availability of data

All manuscripts must include a data availability statement. This statement should provide the following information, where applicable:

- Accession codes, unique identifiers, or web links for publicly available datasets
- A description of any restrictions on data availability
- For clinical datasets or third party data, please ensure that the statement adheres to our policy

The RNA-seq data generated in this study are available at GEO under accession GSE191133 (GEO Accession viewer (nih.gov)). Two published datasets were

reanalyzed in the study (GSE38324: [https://doi.org:10.4161/rna.26921]43 and GSE83432: [https://doi.org:10.1186/s13059-016-1139-1]56). GRCm38/mm10 and GRCh38/hg38 were referenced to sequences alignments in all data. Other data are available in the manuscript or the supplementary materials. Source data are provided with this paper.

Research involving human participants, their data, or biological material

Policy information about studies with human participants or human data. See also policy information about sex, gender (identity/presentation), and sexual orientation and race, ethnicity and racism.

Reporting on sex and gender	N/A
Reporting on race, ethnicity, or other socially relevant groupings	N/A
Population characteristics	N/A
Recruitment	N/A
Ethics oversight	N/A

Note that full information on the approval of the study protocol must also be provided in the manuscript.

Field-specific reporting

Please select the one below that is the best fit for your research. If you are not sure, read the appropriate sections before making your selection.

☒ Life sciences ☐ Behavioural & social sciences ☐ Ecological, evolutionary & environmental sciences

For a reference copy of the document with all sections, see nature.com/documents/nr-reporting-summary-flat.pdf

Life sciences study design

All studies must disclose on these points even when the disclosure is negative.

Sample size	The animal sample sizes were determined according to 3R's of animals used in research (replacement, reduction, and refinement). Besides Supplementary Fig. 4d, all experiments were conducted with at least three independent experiments and multiple biological replicates. Sample sizes were determined as sufficient since they led to similar results. In RNA-seq, three individual clones of CRISPR-Cas9 DNA edited Rncr3 exons 2/3 deletion or Rncr3 full-length deletion in NE-4C cells were used. Three biological replicates of non-manipulated NE-4C cells were used as wild type control.
Data exclusions	Some samples in the Western blot were run twice (shown in Related Source Data), and we ultimately filtered out data that did not show a homogeneous endogenous control.
Replication	Reported results were repeated and confirmed for at least two independent experiments.
Randomization	Randomization is not relevant since in this study all samples were categorized according to their genotypes or drug administrations
Blinding	The immunofluorescent data in cultured cells and mouse brain sections were collected in double-blinded manner. Regarding the other data collection, uniform and consistent standards were performed in those data collection which are not related with subjective judgements.

Reporting for specific materials, systems and methods

We require information from authors about some types of materials, experimental systems and methods used in many studies. Here, indicate whether each material, system or method listed is relevant to your study. If you are not sure if a list item applies to your research, read the appropriate section before selecting a response.

Materials & experimental systems

n/a	Involved in the study
☐	☒ Antibodies
☐	☒ Eukaryotic cell lines
☒	☐ Palaeontology and archaeology
☐	☒ Animals and other organisms
☒	☐ Clinical data
☒	☐ Dual use research of concern
☒	☐ Plants

Methods

n/a	Involved in the study
☒	☐ ChIP-seq
☒	☐ Flow cytometry
☒	☐ MRI-based neuroimaging

Antibodies

Antibodies used

Rabbit polyclonal anti-pH3:Millipore Sigma, #06-570, RRID:AB_310177; Rabbit polyclonal anti-H3:Abcam, #ab1791, RRID:AB_302613; Rabbit polyclonal anti-Cleaved Caspase-3: Cell Signaling, #9661, RRID:AB_2341188; Rabbit polyclonal anti-Tuj1: Sigma-Aldrich, #T3952, RRID:AB_1841226; Rabbit polyclonal anti-Gapdh: Sigma-Aldrich, #G9545, RRID:AB_796208; Mouse monoclonal anti-Map2: Sigma-Aldrich, # M4403, RRID:AB_477193; Mouse monoclonal anti-NFM- anti160kD: Abcam, #ab65845, RRID:AB_1139297; Rabbit polyclonal anti-Ptbp1: Thermo Fisher Scientific, #PA5-81297, RRID:AB_2788516; Rabbit monoclonal anti-Drosha: Cell Signaling Technology, # 3364; RRID:AB_2238644; Rabbit polyclonal anti-Dgcr8: Thermo Fisher Scientific, # PA5-40122; RRID:AB_2606318; Mouse monoclonal anti-BrdU: Molecular Probes, # A-21304; RRID:AB_221472; Mouse monoclonal anti-MeCP2: Active Motif, # 61285; RRID:AB_2572268; Mouse IgG2c, kappa monoclonal [18C8BC7AD10] - Isotype Control antibody: Abcam, # ab170191; RRID:AB_2861163; Rabbit IgG Control Antibody, Unconjugated: Sigma-Aldrich, # I5006; RRID:AB_1163659; Rabbit polyclonal anti-5-Methylcytosine (5-mC): Active Motif, # 61255, RRID:AB_2783884; Rabbit polyclonal anti-eGFP: Thermo Fisher Scientific, # CAB4211, RRID:AB_10709851; Anti- MS2 Coat Protein: Millipore Sigma, # ABE76-I, RRID:AB_2827507

Validation

Rabbit polyclonal anti-pH3: Mouse; Human; ICC, IP, WB, IF
Rabbit polyclonal anti-H3: Mouse, Rat, Human, Saccharomyces cerevisiae, Xenopus laevis, Arabidopsis thaliana, Drosophila melanogaster, Indian muntjac, Schizosaccharomyces pombe; IHC, CHIP, IP, WB, IF;
Rabbit polyclonal anti-Cleaved Caspase-3: Human, Mouse, Rat, Monkey; WB, IP, IF;
Rabbit polyclonal anti-Tuj1: Human, Mouse; WB, IF;
Rabbit polyclonal anti-Gapdh: Mouse; WB;
Mouse monoclonal anti-Map2:rat, chicken, human, mouse, bovine, quail; IF, WB;
Mouse monoclonal anti-NFM- anti160kD: Mouse; IF, WB;
Rabbit polyclonal anti-Ptbp1: Human, Mouse; RIP, IHC, IF, IP;
Rabbit monoclonal anti-Drosha: Human, Mouse; RIP, IF, WB;
Rabbit polyclonal anti-Dgcr8: Amphibian, Human, Mouse; RIP, WB, IF, IP;
Mouse monoclonal anti-BrdU: IF;
Mouse monoclonal anti-MeCP2: Human, Mouse, Rat; RIP, WB, IF, CHIP, IHC, ICC;
Mouse IgG2c, kappa monoclonal [18C8BC7AD10] - Isotype Control antibody: RIP;
Rabbit IgG Control Antibody, Unconjugated: RIP, ELISA, WB;
Rabbit polyclonal anti-5-Methylcytosine (5-mC): Human, Mouse; RIP, CHIP, MeDIP.B, IHC;
Rabbit polyclonal anti-eGFP: WB, RIP, IF;
Anti- MS2 Coat Protein: IP, RIP, WB.

Eukaryotic cell lines

Policy information about cell lines and Sex and Gender in Research

Cell line source(s)

Mouse: NE-4C cells: ATCC #CRL-2925; Human: ReNcell CX immortalized cell line: Millipore SCC007; Human: HEK 293 cells: ATCC #CRL-1573.

Authentication

We have checked all the cell lines use in the study against the list of known misidentified cell lines maintained by the International Cell Line Authentication Committee (https://iclac.org/databases/cross-contaminations/) and they are not on the list.

Mycoplasma contamination

All cell lines were tested negative for mycoplasma contamination

Commonly misidentified lines
(See ICLAC register)

None

Animals and other research organisms

Policy information about studies involving animals; ARRIVE guidelines recommended for reporting animal research, and Sex and Gender in Research

Laboratory animals

Mouse line C57BL/6J: 8-weeks-old to 10-months-old

Wild animals	The study did not involves wild animals.
Reporting on sex	Sex was not considered in this study.
Field-collected samples	The study did not involve the samples collected from the field.
Ethics oversight	All experiments described in this article comply with the relevant ethical regulations and all animal procedures were performed according to animal welfare guidelines and regulations approved by an IACUC approved protocol at the University of Colorado Boulder.

Note that full information on the approval of the study protocol must also be provided in the manuscript.

Plants

Seed stocks	*Report on the source of all seed stocks or other plant material used. If applicable, state the seed stock centre and catalogue number. If plant specimens were collected from the field, describe the collection location, date and sampling procedures.*
Novel plant genotypes	*Describe the methods by which all novel plant genotypes were produced. This includes those generated by transgenic approaches, gene editing, chemical/radiation-based mutagenesis and hybridization. For transgenic lines, describe the transformation method, the number of independent lines analyzed and the generation upon which experiments were performed. For gene-edited lines, describe the editor used, the endogenous sequence targeted for editing, the targeting guide RNA sequence (if applicable) and how the editor was applied.*
Authentication	*Describe any authentication procedures for each seed stock used or novel genotype generated. Describe any experiments used to assess the effect of a mutation and, where applicable, how potential secondary effects (e.g. second site T-DNA insertions, mosiacism, off-target gene editing) were examined.*
